# Deciphering optimal biostimulation strategy of supplementing anthocyanin-abundant plant extracts for bioelectricity extraction in microbial fuel cells

**DOI:** 10.1186/s13068-019-1385-z

**Published:** 2019-03-01

**Authors:** Bin Xu, John Chiwei Lan, Qingjiang Sun, Chungchuan Hsueh, Bor-Yann Chen

**Affiliations:** 1 State Key Laboratory of Bioelectronics, School of Biological Science & Medical Engineering, and Research Center for Learning Science, Southeast University, Nanjing, 210096 People’s Republic of China; 2 Department of Chemical and Materials Engineering, National I-Lan University, Yilan, 26047 Taiwan; 3 Biorefinery and Bioprocess Engineering Laboratory, Department of Chemical Engineering and Materials Science, Yuan Ze University, Chungli, Taoyuan, 32003 Taiwan

**Keywords:** Anthocyanin, Microbial fuel cells, Redox mediators, Polyphenolic antioxidants, Electron transport chain, Adenosine triphosphate synthesis

## Abstract

**Background:**

Microbial fuel cells (MFCs) are effective biofuel devices that use indigenous microbes to directly convert chemical energy from organics oxidation into bioelectric energy. To maximize energy-converting efficiency for bioelectricity generation in MFCs, redox mediators (RMs) (e.g., extracts obtained from plant resource-*Camellia* green tea) have been explored for optimal stimulation upon electron transfer (ET) capabilities. Anthocyanins are natural antioxidants widely used in food science and medicinal industry. This first-attempt study revealed optimal strategies to augment extracts of anthocyanin-rich herbs (*Lycium ruthenicum* Murr., *Clitoria ternatea* Linn. and *Vaccinium* Spp.) as biofuel sources of catalytic RMs for stimulating bioenergy extraction in MFCs.

**Results:**

This work showed that extracts of anthocyanin-rich herbs were promising electroactive RMs. The maximal power density of MFCs supplemented with extract of *L. ruthenicum* Murr. was achieved, suggesting that extract of *L. ruthenicum* Murr. would be the most electrochemically appropriate RMs. Compared to *C. ternatea* Linn. and *Vaccinium* Spp., *L. ruthenicum* Murr. evidently owned the most significant redox-mediating capability to stimulate bioenergy extraction likely due to significantly high contents of polyphenols (e.g., anthocyanin). Evidently, increases in adenosine triphosphate (ATP) content directly responded to supplementation of anthocyanin-rich herbal extracts. It strongly suggested that the electron-shuttling characteristics of RMs upon electroactive microorganisms could effectively promote the electron transfer capability to maximize bioenergy extraction in MFCs.

**Conclusion:**

Anthocyanin as the main water-soluble vacuolar pigments in plant products were very electroactive for not only excellent antioxidant activities, but also promising electron-shuttling capabilities for renewable biofuel applications. This work also suggested the electron-shuttling mechanism of RMs that could possibly promote electron transport phenomena through microbial cell membrane, further influencing the electron transport chain for efficient bioenergy generation.

## Background

For sustainable development, biomass-based energy was considered to be one of the most appropriate renewable energy due to its environmental friendliness. Among diverse sources of bioenergy, MFCs as electrochemical system directly convert the chemical energy of organic matter into electrical energy with the assistance of electrochemically functioning bacteria [1–3]. In addition, using MFCs as electrochemically steered bioreactors, the performance of pollutant biodegradation could be significantly augmented as well [4–7]. To increase the rate of redox reaction for energy extraction, exogenous augmentation of RMs could also reduce electron transfer resistance, considerably augmenting power generation in MFCs. Recent findings [8–10] also revealed that naturally biosynthesized electron shuttle-augmented MFC was electrochemically favorable for green bioenergy extraction due to effective ET stimulation of such naturally generated RMs. For example, Chen et al. [11] used edible flora and medicinal herbs as possible RM sources to effectively stimulate power generation in MFCs. As electrochemical catalysts, RMs can reversibly transfer electrons between oxidized species and reduced species, mediating ET phenomena for efficient energy utilization (e.g., catechol, vitamin B_2_-riboflavin) [12]. With the augmentation of RMs for bioelectrochemically feasible bioenergy extraction, such ET-stimulating capabilities could simultaneously enhance effective waste degradation and energy-recycling utilization. Furthermore, to avoid introduction of artificially synthesized RMs as secondary contaminant(s), supplementing naturally generated RMs would be one of top priority alternatives to catalytically facilitate sustainable bioenergy extraction. As cyclic voltammetric analyses indicated [13–15], hydroxyl substituent(s) were likely to be more electrochemically stable and reversible than amino groups to augment ET phenomena. Considering hydroxyl substituent-bearing environmental resource(s), polyphenolics-abundant plant products could be electrochemically applicable as green RMs to simulate BG for bioenergy extraction [16]. Although Chen and Hsueh [17] proposed first-attempt approaches with electrochemical evaluation to screen some extract of herbal medicine and edible vegetables as feasible RMs for stimulating bioenergy recycling (e.g., MFCs, electro-fermentation), detailed mysteries behind such electrochemical catalysis (e.g., operation strategy to reversibly convert capabilities of polyphenolic antioxidants to sustainable RMs) have still not been uncovered for industrial practicability.

**Fig. 1:**
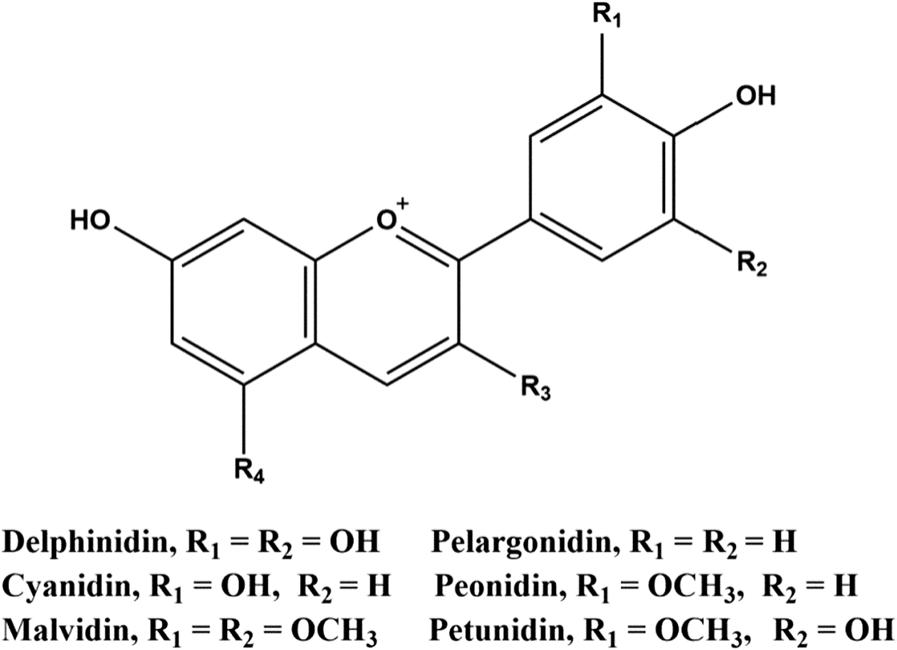
Basic chemical structures of the major classes in anthocyanidin

Apparently, the food industry and medicinal branches are paying significant attentions to plant polyphenols more than ever due to myriads of functions in antioxidant activity, antimicrobial, antiviral, and anti-inflammatory properties [18–21]. Polyphenolic compounds were crucial secondary metabolites in plants to prevent free radicals and oxidative stress from the environment. These compounds also contribute to the color and sensory characteristics (e.g., electron resonant capabilities) of fruits and vegetables. Polyphenols could be broadly classified in four classes: phenolic acids, flavonoids, stilbenes, and lignans [18, 20]. In particular, flavonoids are the most abundant polyphenols in daily diet to humans. As one of the most significant subclasses of phenolic compounds in the parent class of flavonoids, anthocyanin is the most important water-soluble vacuolar pigments regularly synthesized via phenylpropanoid pathway [18, 20]. However, anthocyanin is a diphenylpropane-based polyphenolic ring structure, and is limited to a few structure variants including delphinidin (Dp), pelargonidin (Pg), cyanidin (Cy), peonidin (Pn), petunidin (Pt), and malvidin (Mv) (Fig. 1) [20]. In addition, *R*_3_ and *R*_4_ are always connected with glucoside to form macromolecular structure (e.g., cyanidin-3-*O*-glucoside and malvidin-3,5-di-*O*-glucoside). These all are responsible for color-generating capabilities in most fruits, vegetables, flowers, and some cereal grains. The most common anthocyanidin glycosides were 3-glycosides, and thus, the most widespread anthocyanin was cyanidin 3-glucoside [22]. Different antioxidant properties of individual anthocyanin were related to the number and position of hydroxyl groups in aromatic rings, and also the characteristics, number, and position of glucoside attached to the molecule [20]. These structural differences will also lead to the differences in properties of anthocyanin (e.g., the color, stability, and antioxidant activity). In addition, due to environment-sensitive characteristics, anthocyanin could be present in different chemical forms and emerged different physicochemical properties with a change of solvent or pH [20, 23]. Therefore, due to natural abundance, environmental friendliness and diverse applicability, the role of anthocyanin as alternative to replace artificial colorants is increasingly the focus of worldwide attention. The most significant function of anthocyanin is the ability to impart color to the plants or plant products for applications. Compared to artificially synthesized pigment, anthocyanin is naturally produced, potentially harmless, and water-soluble in aqueous media, making them compelling for practical uses as food colorants [24, 25]. Apart from the potential to be natural food pigments, anthocyanin was also applicable to be medicine for treatment of diabetes, eyesight disorders or coronary diseases. These were likely associated to the antioxidative action as free radical scavengers. For example, towards type II diabetes, Sancho and Pastore [26] suggested that anthocyanin may lower blood glucose by improving insulin resistance, protecting β cells, increasing secretion of insulin and reducing digestion of sugars in the small intestine due to significant antioxidant characteristics. As Fan et al. [27] revealed, anthocyanin from black rice (*Oryza sativa*) could promote immune responses to leukemia through enhancing phagocytosis of macrophages in vivo. Furthermore, Singletary et al. [28] indicated that anthocyanin-rich extract from grape have chemopreventive potential to breast cancer possibly due to their capacities to block carcinogen–DNA adduct formation and modulate activities of carcinogen-metabolizing enzymes. Wang and Stoner [29] also concluded that the antioxidant effects of anthocyanin in vitro have been demonstrated using several organ cell culture systems. Our recent findings [10, 11, 16, 30] also suspected that the efficacy of medicinal herbs was possibly associated with electrochemical activities (e.g., antioxidant and RM activities). Aside from the health benefits, anthocyanin was also applied to electrochemical applications (e.g., smart electronic devices, sensor, and fuel cells). Amiri-Aref et al. [31] utilized a bioactive anthocyanin for the fabrication of a novel carbon nanotube-bearing sensor for selective determination of l-DOPA (one kind of *ortho*-dihydroxyl substituents-bearing RMs) in the presence of uric acid. Furthermore, San Esteban and Enriquez [32] used composite of anthocyanin with graphene as photosensitizer to augment efficiency of dye-sensitized solar cells. This first-attempt study tended to quantitatively evaluate electron-shuttling characteristics of anthocyanin to stimulate biomass energy extraction for practical applications. In particular, this feasibility study also revealed maximal conversion of reversible antioxidant compositions to be electrochemically catalytic RMs for sustainable bioenergy extraction in MFCs.

As prior study indicated [13], electron-shuttling functional groups [e.g., dihydroxyl (−OH) substituents] present in the *ortho* or *para* position of benzene ring could significantly exhibit stable and reversible electron-shuttling characteristics. In fact, considering anthocyanins ubiquitous in nature, delphinidin (Dp) and cyanidin (Cy) apparently satisfied such feature of chemical structure. This work explored whether antioxidant anthocyanin could also act as electrochemically catalytic RMs to stimulate bioelectricity generation in MFCs. Three model anthocyanin-related plants were intentionally selected as test natural bioresource for feasibility study. As this study revealed, both significant reductive and oxidative peak currents were shown in cyclic voltammetric profiles. Their promising free radical-scavenging capabilities of plant extract strongly suggested that anthocyanin evidently owned remarkable electron-shuttling potentials for bioenergy applicability. This study also further confirmed such electron transfer-stimulating phenomena to augment bioelectricity generation in MFCs for encouraging feasibility. To provide supporting evidence for effective redox-mediating capabilities, transient dynamics of ATP expression with the supplementation of anthocyanin was also revealed in MFC module reactors. Likely due to increased contents in anthocyanin, amplified electron-shuttling capabilities increasingly assisted electrons transferring on cellular surface between intracellular compartment and membrane-bound electron transport chain, resulting in increases in ATP production. That is, a substantial reduction of electron transfer resistance led to an increase in the rate of bioelectricity generation and the production of target metabolite(s). This study suggested a renewable alternative to provide natural RM chemicals to drastically reduce electron-transfer resistance and to maximize power-generating capabilities in MFCs for sustainable bioenergy extraction.

## Results and discussion

### Redox-mediating capability assessment

**Fig. 2:**
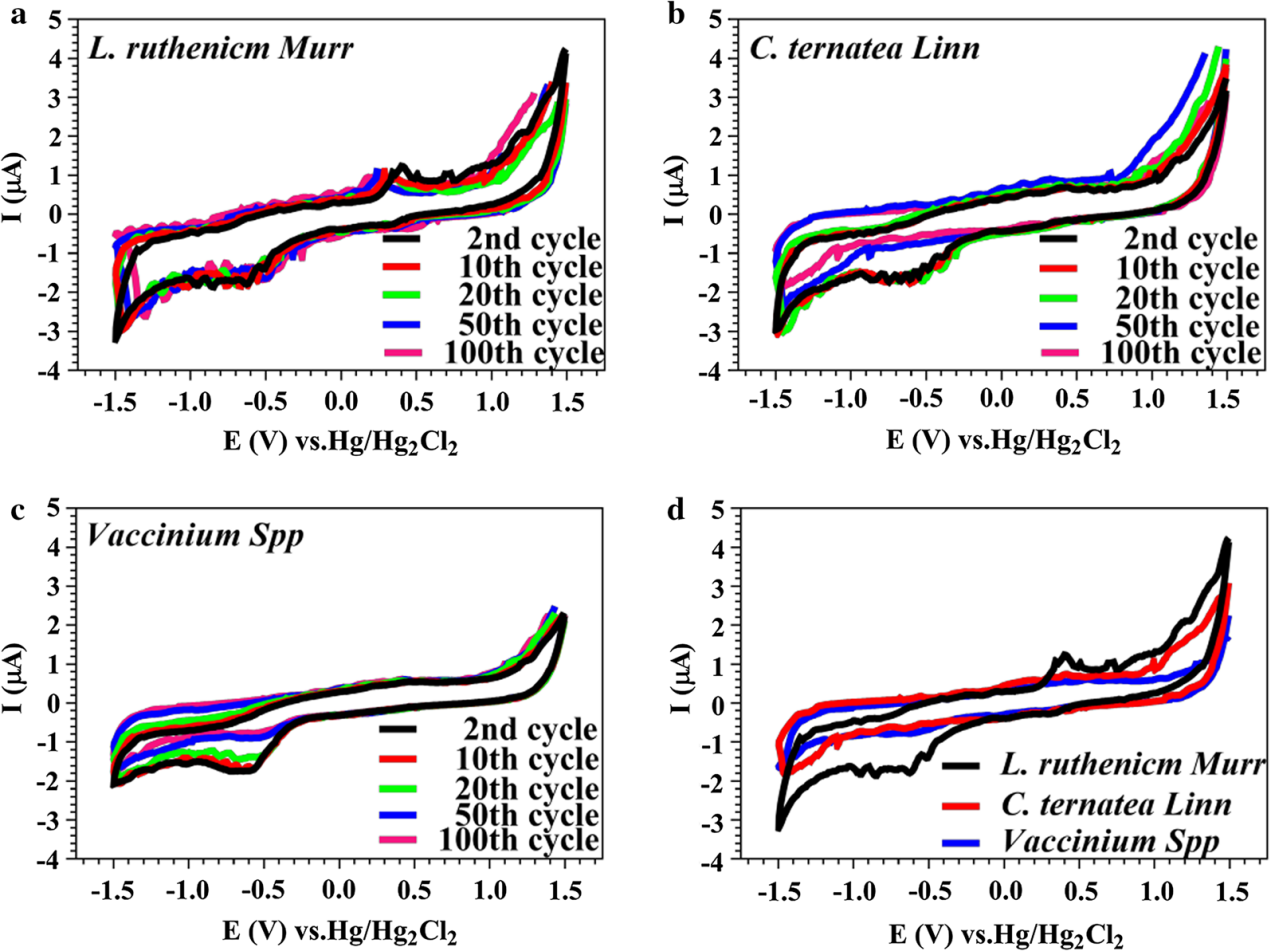
Comparison of CV profiles of **a** *L. ruthenicm* Murr., **b** *C. ternatea* Linn., **c** *Vaccinium* Spp. and **d** overall comparison for the 100th cycle of CV profiles at the concentration of 1000 mg L^−1^

To explore electrochemical characteristics of antioxidants and RMs, cyclic voltammetric (CV) analysis of model anthocyanin-containing plant extracts was quantitatively implemented. Prior studies [13, 14] indicated that artificially synthesized aromatic chemicals (e.g., anthraquinones and textile dyes) could act as RMs to stimulate performance of simultaneous wastewater decolorization and bioelectricity generation of microbial fuel cells (MFCs). Recently, for green sustainability, natural edible plant extracts (e.g., *Camellia* green tea and refreshing medicinal herbs) were supplemented to MFCs, significantly augmenting power-generating capabilities even after several times of extraction (e.g., after 5 times serial brewing, *Camellia* green tea still had ca. 50% residual activity) [30]. When multiple cycle cyclic voltammetric profiles of herbal extracts still stably exhibited both reductive and oxidative potential peaks, these extracts apparently contained not only non-renewable antioxidant species, but also sustainable RMs. To explore whether anthocyanins own electron-shuttling capability, CV scanning of anthocyanin-abundant extracts (i.e., *L. ruthenicm* Murr., *C. ternatea* Linn. and *Vaccinium* Spp.) was implemented for comparative analysis (Fig. 2). To clearly indicate such quasi-reversibility of electron-shuttling characteristics of RMs, multiple cycle CV profiles (i.e., continuous responses to serial electrochemical oxidation and reduction) were at least carried out for 100 cycles. As shown in Fig. 2a, apparently the extract of *L. ruthenicm* Murr. expressed the most electrochemically stable redox potential peaks and electrochemical profile of CV scanning compared to two other extracts. In contrast, significant decay of CV profiles in Fig. 2b, c suggested that substantial contents of antioxidants and/or anti-reductants were electrochemically utilized as similarly indicated in prior studies for herbal extracts of *Syzygium aromaticum*, *Lonicera japonica* [30]. This phenomenon could also be quantitatively revealed through determination of the closed-loop area of CV profiles (Table 1). Apparently, after ca. ten cycles of CV scan-in-series, the closed-loop area of *L. ruthenicm* Murr. asymptotically achieved the most electrochemically stable value. The most reversible and stable CV profiles of *L. ruthenicm* Murr. also suggested that this plant extract contained the most promising electrochemical species (e.g., antioxidants and RMs) to be utilized for sustainable and renewable energy. Two other extracts still appreciably exhibited gradual attenuation of electrochemical activities. Comparative analysis upon the area of 100th cycle of CV scan, the ranking (unit: V μA) of the three anthocyanin-rich extracts at 1000 mg L^−1^ was *L. ruthenicm* Murr. (4.57) > *C. ternatea* Linn. (3.46) > *Vaccinium* Spp. (2.97), indicating that among these model anthocyanin-rich extracts *L. ruthenicm* Murr. owned the most promising electrochemical activity to stimulate more effective power generation in MFCs. Regarding minor redox peaks as shown in Fig. 2, although pure substance was chosen for CV scan, slightly different shapes of CV cumes (e.g., some minor redox peaks) would still appear. In addition, these were also strongly dependent upon modes of CV scanning [13]. However, major redox potential peaks as disclosed herein were still remained nearly identical as fingerprint characteristics. That is, scanning rate would not significantly alter the fingerprint characteristics of redox mediator(s) to be tested. Therefore, minor peaks shown in CV profiles may be simply considered as background noises and they should not significantly affect predominant electrochemical characteristics of test RMs.

**Table 1:** Comparative list of closed-loop area^a^ of CV scanning profiles (unit: V μA)

	Cycle 2	Cycle 10	Cycle 20	Cycle 50	Cycle 100
*L. ruthenicm* Murr.	5.60	4.92	4.66	4.58	4.57
*C. ternatea* Linn.	5.32	5.24	4.56	3.83	3.46
*Vaccinium* Spp.	4.21	4.09	3.75	3.16	2.97

^a^${\text{Area}} = \mathop \smallint \nolimits_{{V_{\text{L}} }}^{{V_{\text{H}} }} \left( {i_{\text{h}} - i_{\text{l}} } \right){\text{d}}V$. The parameters *V*_H_, *V*_L_ represented the CV scanning voltages of + 1.5 V and − 1.5 V at cycle *n*, respectively; *i*_h_, *i*_l_ denoted the oxidation and reduction currents at specific scan voltage, respectively

### Antioxidant activity evaluation

**Fig. 3:**
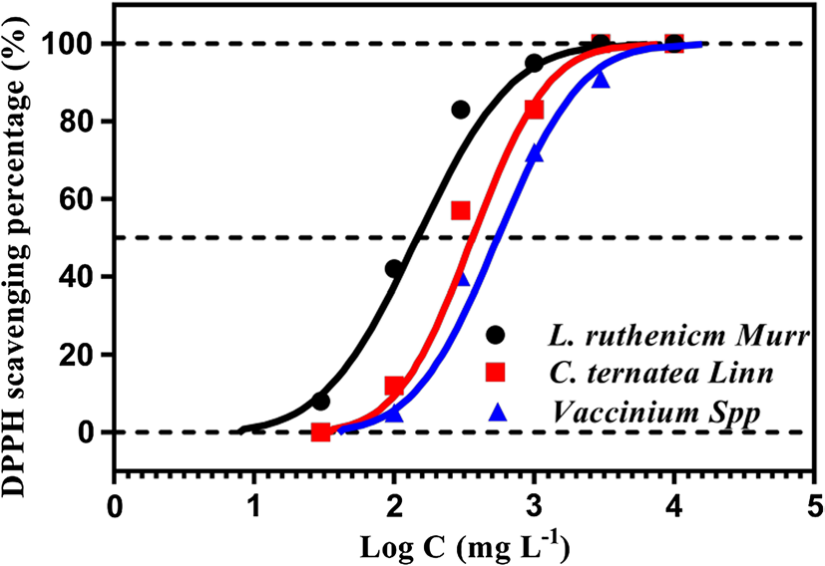
Comparative dose–response curves of antioxidant capabilities of anthocyanin-rich extracts

As Chen et al. [10] proposed, total phenolic content, antioxidant activity and electron-shuttling capability seemed to be statistically associated for polyphenol-abundant herbal extracts as they were all strongly associated with electrochemical activities. Capabilities of RMs could also be positively proportional to antioxidant activity compared to non-polyphenol-rich antioxidants. To assess the antioxidant activity of anthocyanin-rich extracts, DPPH free radical scavenging capabilities of *L. ruthenicm* Murr., *C. ternatea* Linn. and *Vaccinium* Spp. were comparatively assessed (Fig. 3). For comparative assessment, the antioxidant activity of test samples could be illustrated in dose–effect relationships by the parameters of effective concentration (EC_*x*_), which indicated an *x*% removal response of DPPH free radicals [33]. EC_0_ was defined as the threshold concentration to have a detectable response of antioxidant activity. In contrast, EC_100_ is the minimal concentration of 100% response of antioxidant. EC_50_ means the half maximal effective concentration. For comparative ranking, the more severe the antioxidant activity measured, the lower the EC_50_ for the DPPH free radical-scavenging activity. All of the critical parameters EC_*x*_ were listed in Table 2 for quantitative evaluation. The smaller EC_*x*_ value and the far left-side curve of test herbal sample simply suggested more severe antioxidant activity to be exhibited. As indicated in Fig. 3 and Table 2, the ranking of antioxidant activity based on EC_0_ and EC_50_ (unit: mg L^−1^) was both followed the identical series: (1) EC_0_: *L. ruthenicm* Murr. (6.76) < *C. ternatea* Linn. (28.18) < *Vaccinium* Spp. (35.47); (2) EC_50_: *L. ruthenicm* Murr. (125.89) < *C. ternatea* Linn. (295.12) < *Vaccinium* Spp. (478.63). These results all indicated that *L. ruthenicm* Murr. owned the highest antioxidant activity among three anthocyanin-rich extracts. It was also suspected that the ranking of electron-shuttling capabilities was made in parallel to the order for anthocyanin-rich plant extracts to stimulate bioelectricity generation in MFCs. This finding reflected well on the CV results. That is, antioxidant capability was essentially in agreement with the electron-shuttling activity due to strong electrochemical association between antioxidant activity and RM capability. To directly assess the relationship between dose and effect intensity of these anthocyanin-rich plant extracts, the equation of linear regression (i.e., *Y* = *B* log *Z* + *A*) was adopted to reflect the antioxidant ability of “acute responses” [34]. In addition, the slope factor *B* in dose–effect relationships (Table 2) was also calculated to represent test samples were elucidated in either the “acute” or “chronic” mode of antioxidant activity. In general, the unity of slope factor *B* in dose–response curves was usually used to classify acute or chronic responses of test substance. The steeper curves with greater slope factor *B* clearly suggested more electrochemically promising antioxidant capabilities of anthocyanin-rich herbal extracts. As indicated in Table 2, all of slope factor *B* values greater than unity simply suggested that all test substances were electrochemically effective to significantly respond the antioxidant activity.

**Table 2:** Key parameters comparison on dose–response curves of antioxidant capabilities (unit: mg L^−1^)

	EC_0_	EC_50_	*Y* = *B* log*Z* + *A*	*B*
*L. ruthenicm* Murr.	6.76	125.89	*Y* = 1.88 log*Z* + 1.0384	1.88
*C. ternatea* Linn.	28.18	295.12	*Y* = 2.34 log*Z* − 0.8025	2.34
*Vaccinium* Spp.	35.47	478.63	*Y* = 2.12 log*Z* − 0.6882	2.12

### Total polyphenols content analysis

As aforementioned, herbal extracts of anthocyanin-rich plants (i.e., *L. ruthenicm* Murr., *C. ternatea* Linn., and *Vaccinium* Spp.) all significantly exhibited similar rankings of electrochemical ES activity and antioxidant activity. As proposed in Chen and Hsueh [17], considering sustainable catalysis of RMs, the more stably reversible electron-shuttling functional substituent(s) would be hydroxyl group(s) compared to less electrochemically reversible amino-group(s). In fact, according to Przygodzka et al. [35], antioxidant activity was likely attributed to contents of polyphenolics and flavonoids. Therefore, the strong electron-shuttling and antioxidant activity of test anthocyanin-rich herbal extracts might be due to a substantial content of total polyphenolics in extracts. As suggested in Table 3, the TPC ranking (unit: mg GAE g^−1^ EW) of model anthocyanin-rich extracts was *L. ruthenicm* Murr. (103.11 ± 4.83) > *C. ternatea* Linn. (75.87 ± 3.66) > *Vaccinium* Spp. (56.12 ± 3.18). The parallel rankings between CV profiles and DPPH free radical scavenging capability were clearly revealed. In fact, using quercetin as the standard equivalent, Jiang et al. [36] obtained the TPC of *L. ruthenicm* Murr. at 1555 ± 112 mg quercetin equivalent/100 g freeze dry powder. Lee and Wrolstad [37] also quantified the TPC of *Vaccinium* Spp. at 1583 mg/100 g (gallic acid equivalents). As the dry powders of anthocyanin-rich herbal extracts used herein were highly concentrated prior to the freeze drying process, higher concentration of total polyphenol and anthocyanin could thus be obtained. Although anthocyanin could be present in different chemical structures due to different substituent groups, to have conclusive remarks the main perspectives of this study were focused on those anthocyanins with dihydroxyl (−OH) in the chemical structures, in particular for delphinidin (Dp), cyanidin (Cy) and petunidin (Pt), as indicated in Fig. 1. The bioenergy-stimulating effects of these three chemical structures could be quantitatively compared with the basic structure of gallic acid (GA) for conclusions. Therefore, TPC could simply represent the actual anthocyanin content in the anthocyanin-containing plant extracts to a reasonable extent with GA as the standard equivalent.

**Table 3:** Comparison on TPC of anthocyanin-rich extracts (unit: mg GAE g^−1^ EW^a^)

Anthocyanin-rich extracts	TPC
*L. ruthenicm* Murr.	103.11 ± 4.83
*C. ternatea* Linn.	75.87 ± 3.66
*Vaccinium* Spp.	56.12 ± 3.18

^a^GAE and EW denoted GA equivalent and extract weight, respectively

### Bioelectricity-stimulating capability analysis

**Fig. 4:**
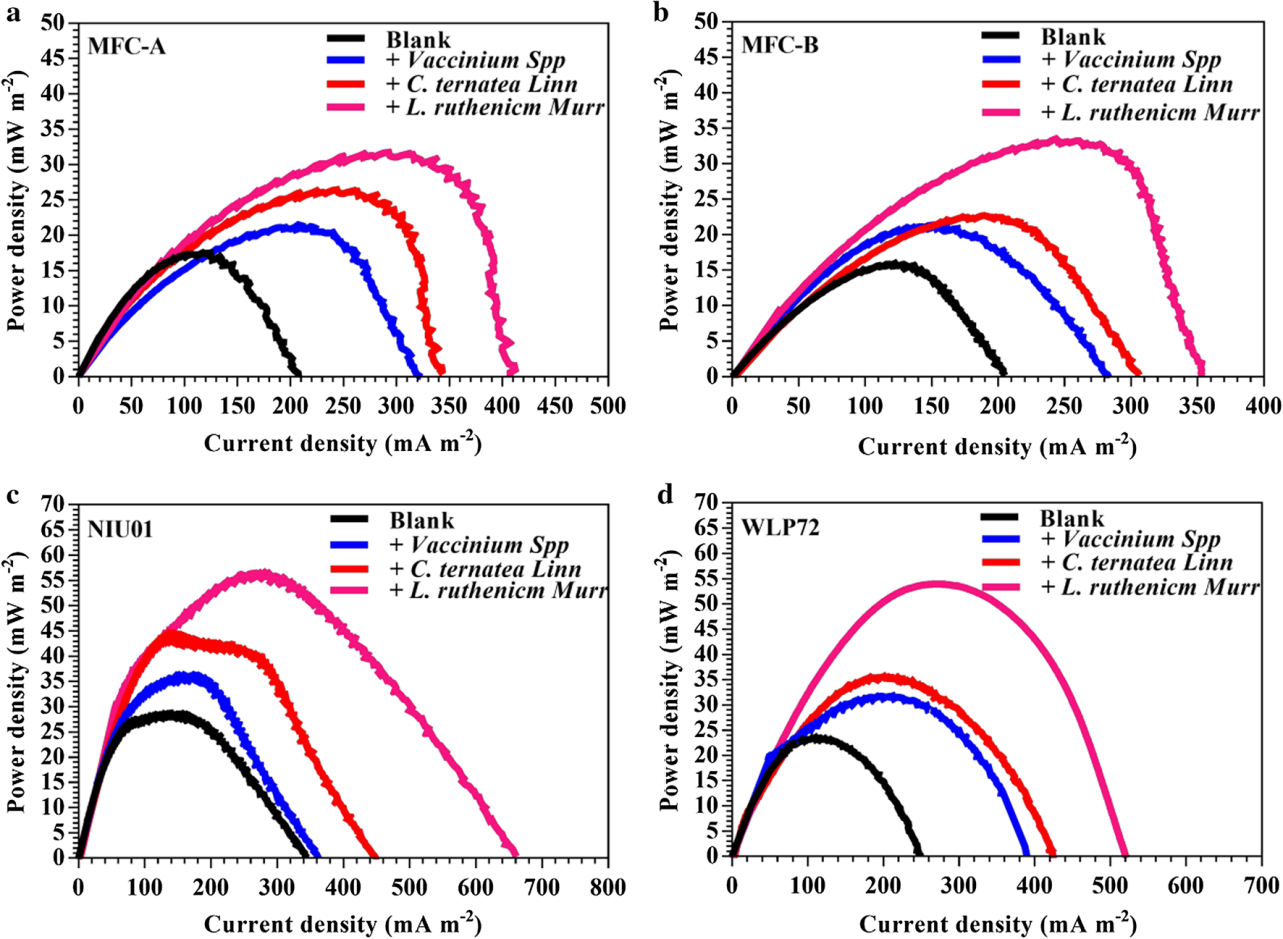
Comparison on power density curves of mixed consortia-seeded microbial fuel cells **a** MFC-A, **b** MFC-B and pure bacterium-inoculated microbial fuel cells **c** NIU01, **d** WLP72 with supplementation of *L. ruthenicm* Murr., *C. ternatea* Linn. and *Vaccinium* Spp. extracts

Although both antioxidant activity and electron-shuttling activity were electrochemically associated and positively proportional to each other, both capabilities were still measured in the absence of receptor microbes. Thus, to grasp whether such activities could be fully biologically expressed, bioelectricity stimulating capability using MFC modules was required to be quantitatively assessed. To reveal whether anthocyanin could act as exogenous RMs, quantitative comparison upon power density-profiles of MFCs supplemented with different anthocyanin-rich extracts (i.e., *L. ruthenicm* Murr., *C. ternatea* Linn. and *Vaccinium* Spp.) was conducted. In fact, mixed consortia and pure bacterium-inoculated MFCs were both carried out to ensure whether the expression of electrochemical RMs in MFCs still depend on the electroactive-bacterial species (Fig. 4). As shown in Table 4, the power density performance of both mixed consortia MFCs and pure bacterium-inoculated MFCs with supplementation of these anthocyanin-rich extracts was revealed. Apparently, significant increases in power density could be observed after supplementation of model extracts (Fig. 4) compared to the blank for both mixed culture and pure bacterium-inoculating double chamber (DC)-MFCs. The ranking of maximum power density (unit: mW m^−2^) of these MFCs was (a) MFC-A: *L. ruthenicm* Murr. (31.72) > *C. ternatea* Linn. (26.43) > *Vaccinium* Spp. (21.68) > blank (17.56); (b) MFC-B: *L. ruthenicm* Murr. (33.64) > *C. ternatea* Linn. (22.97) > *Vaccinium* Spp. (20.93) > blank (15.84); (c) NIU01: *L. ruthenicm* Murr. (56.42) > *C. ternatea* Linn. (44.81) > *Vaccinium* Spp. (36.21) > blank (20.77); (d) NIU01: *L. ruthenicm* Murr. (54.37) > *C. ternatea* Linn. (35.91) > *Vaccinium* Spp. (31.78) > blank (23.51). All of mixed culture and pure bacterium-bearing MFCs simply exhibited significant improvement. The ranking in power density with supplementation of extracts was *L. ruthenicm* Murr. > *C. ternatea* Linn. > *Vaccinium* Spp. For anthocyanin-abundant plant extracts, bioelectricity-stimulating activity was strongly associated to total phenolic content. Although there might be some other combined interactions that could still affect electron transfer synergistically or antagonistically, such strong effect related to polyphenols content was experimentally confirmed/verified in parallel with first proposed concept in Chen et al. [30]. Moreover, to maximize the efficiency of bioenergy extraction, the factors to affect responses of electrochemical activities at least included the concentration–time relationships in cell compartments, mass transfer resistance for routes of exposure, physical and chemical characteristics of candidate ES substance(s), biochemical interactions, variations of combined interactions in abiotic and biotic phases. This study provided first-attempt to suggest possible utilization of non-sustainable antioxidants to be renewable ES species for sustainable development. The efficiency of the test ES substance(s) to stimulate bioenergy extraction is the net result of the above-mentioned combined interactions among all of these factors.

**Table 4:** Comparison on maximum power density of mixed consortia-seeded MFCs (unit: mW m^−2^) and pure bacterium-inoculated MFCs (unit: mW m^−2^)

	Blank	*L. ruthenicm* Murr.	*C. ternatea* Linn.	*Vaccinium* Spp.
(a)				
MFC-A	17.56	31.72	26.43	21.68
MFC-B	15.84	33.64	22.97	20.93
(b)				
NIU01	20.77	56.42	44.81	36.21
WLP72	23.51	54.37	35.91	31.78

### Electron-shuttling mechanism

Prior studies [13] have indicated that aromatic compounds with electron-shuttling functional groups [e.g., *ortho*- or *para*-dihydroxyl (−OH) substituent(s)] could act as RMs to enhance bioelectrogenesis in MFCs. Chen et al. [9] first proposed the mechanisms of inter-conversion between the oxidative and reductive state of candidate RMs. Thus, RMs could be reversibly inter-converted between reduced and oxidized forms of intermediates to enhance electron transfer phenomena between electron donor(s) and electron acceptor(s) for augmenting electricity generation. However, in MFC system, detailed mechanism of electron-shuttling between RMs and electroactive bacteria was still remained open to be explored. As Logan and Regan [38] indicated, the performance of bioelectricity production was most likely controlled via three main mechanisms, which were all connected with the generation and transmission of electrons. Strycharz et al. [39] also showed the scheme of electron transfer mechanism through an electroactive anodic biofilm, which also confirmed the Logan’s findings. However, these points were not specifically emphasized on the direct connections with RMs and electroactive bacteria. Thus, this first-attempt study also tended to propose such scheme of electron transfer mechanism between RMs and microbial cells. In fact, prior study [40] explored the electron-shuttling capabilities of aromatic compounds (e.g., 1-amino-2-naphthol and 4-amino-1-naphthol) to stimulate electro-fermentation performance of echinenone bioproduction. The finding also indicated that supplementation of RMs to fermentation system could significantly augment electron transport efficiency of microorganisms to effectively utilize carbon and energy nutrient sources and produce target product(s).

**Fig. 5:**
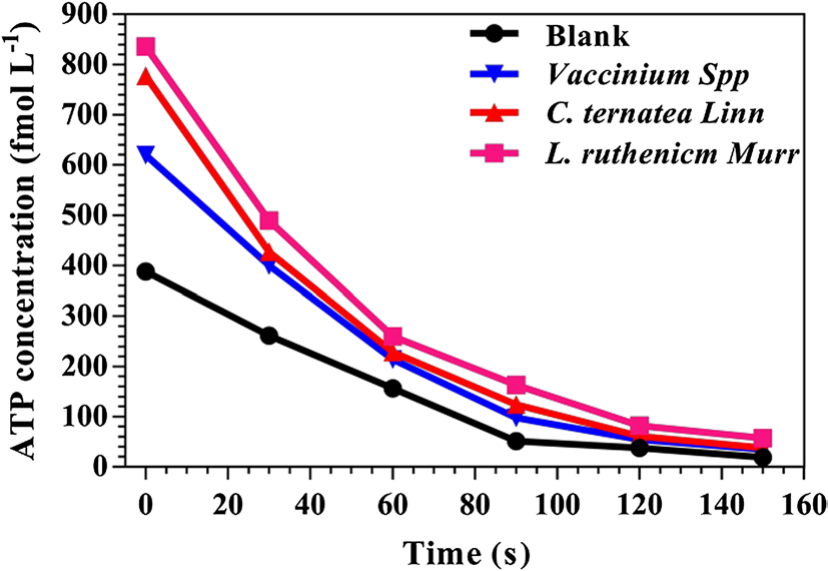
Comparative profiles of ATP synthesis with the supplementation of anthocyanin-rich extracts in pure bacterium-inoculated double chamber MFCs

**Fig. 6:**
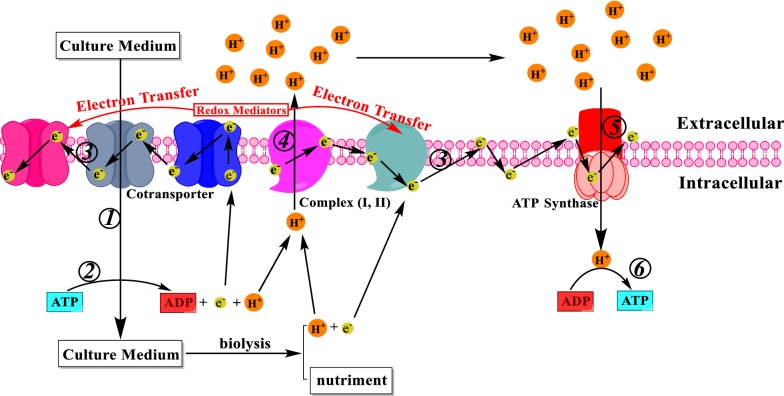
Schematic diagram of mechanisms between redox mediators and electroactive bacteria in MFCs

Adenosine triphosphate (i.e., a complex organic chemical that provides energy for cellular metabolism) as the “bioenergy currency” could intracellularly store and release energy through the mutual conversion to adenosine diphosphate (ADP) and adenosine monophosphate (AMP), ensuring the energy supply persistently provided for metabolic processes in cells. Since electron transport could lead to proton flow coupled to ATP production across cell membrane, this study also explored transient dynamics of ATP concentration when anthocyanin-rich extracts were augmented in pure bacteria-seeded MFCs. Four pure bacteria-bearing MFCs were all seeded with identical bacterial strain (i.e., dye-decolorizing bacterium *Aeromonas hydrophila* NIU01) to ensure data reproducibility and to prevent confounding interactions of different bacterial species. As indicated in Fig. 5, with the supplementation of *L. ruthenicm* Murr., *C. ternatea* Linn. and *Vaccinium* Spp., apparently ATP concentration would directly increase due to stimulating phenomena for energy extraction compared to the blank. That is, anthocyanin-rich extracts could stimulate metabolic activities (e.g., oxidative phosphorylation for ATP production) in cells to generate and store bioenergy in the form of ATP. Compared to stimulation effect of anthocyanin-rich extracts, the ranking of ATP concentration (unit: fmol L^−1^) also followed the series as aforementioned: *L. ruthenicm* Murr. (836) > *C. ternatea* Linn. (777) > *Vaccinium* Spp. (620) > blank (388). This might suggest that the stimulation of electron-shuttling capability could strongly influence the synthesis of ATP for simultaneous responses to energy supply-and-demand in cells. The detection mechanism of ATP synthesis was based on luciferin/luciferase reaction [i.e., (with firefly luciferase] d-luciferin + ATP + O_2_ → oxyluciferin + PPi + AMP + CO_2_ + hν (560 nm)]. Once the reaction took place in the detection bar (or microplate reader) with fluorescein, there would be a proportional loss in the amount of ATP, leading to the decrease of indicator cellular signaling. That was why the data was revealed in a continuous decline and of course initial rate would be crucial to exhibit the content of stored metabolic energy. That is, the comparative data of 150 s to emphasize that such bioenergy-stimulating phenomena upon ATP synthesis were not occasional. As proposed in Fig. 6, culture medium (i.e., the source of nutrient substrates) should be transported into cellular compartment via channel proteins (e.g., Cotransporter) (refer to ① in Fig. 6) [41]. During this process, ATP would be the energy donor to support endocytosis and further been transformed into ADP (refer to ② in Fig. 6) [42]. Theoretically, the release of energy was also accompanied by generation of electrons and protons from the consumption of ATP (i.e., ATP → ADP + H^+^ + e^−^) [43]. In addition, the biological decomposition of culture medium would also provide electrons and protons from the consumption of energy nutrient (or nutriment). As indicated in Xu et al. [40] for the prior postulated mechanism, electrons could transferred through channel proteins on cell membrane and shuttled among these proteins (refer to ③ in Fig. 6), leading to a substantial difference in electrochemical potential between inside and outside of the cell membrane to reduce electron transfer resistance between cytoplasmic compartment and extracellular medium. Such potential difference could act as a driving force to increase the activity of channel proteins to accelerate transmission through cell membrane (refer to ①, ④, and ⑤ in Fig. 6), in particular the proton gradient for transfer. The protons were transferred backward to the cells by channel proteins (i.e., ATP synthase) and participate in the re-synthesis of ATP (refer to ⑥ in Fig. 6). This process was so-called the electron transport chain. The supplementation of anthocyanin-rich extracts was likely to accelerate electron transfer characteristics upon cell membrane to shuttle between intercellular compartment and extracellular medium. This finally led to such increases of ATP concentration. In fact, the faster consumption of carbon source-sucrose after supplementation of RMs for electro-fermentation [40] might also indirectly confirm this proposed mechanism.

## Conclusion

Anthocyanin-rich extracts (i.e., *L. ruthenicm* Murr., *C. ternatea* Linn. and *Vaccinium* Spp.) as effective RMs could exhibit significant electrochemical activities to enhance power-generation in MFCs. Compared to *C. ternatea* Linn. and *Vaccinium* Spp., apparently *L. ruthenicm* Murr. owned the most significant redox-mediating capability and favorable antioxidant activity due to significantly higher content of polyphenols (i.e., anthocyanin). Significant increases of ATP concentration caused by supplementation of anthocyanin-rich extracts also supported the stimulation of electron-shuttling characteristics of RMs. RMs could possibly promote the electron transfer on cell membrane, which further influenced the electron transport chain. Thus, bioelectricity-generating capabilities in MFCs were increased (ca. 1.8 and 2.7 fold for *L. ruthenicm* Murr.).

## Methods

### MFC construction

Regarding double chamber-MFCs, contact areas of the graphite (Grade: IGS743; Central Carbon Co. Ltd.) anode and cathode with cultured broth or electrolyte solutions were ca. 0.001649 m^2^ [i.e., (*π* × 0.5^2^ + 2*π* × 0.5 × 5.0) × 10^−4^]. The cathodic and anodic chamber (working volume 200 mL) were separated by proton exchange membrane (DuPontTM Nafion^®^ NR-212) in contact area ca. 0.000452 m^2^ (ID = 1.2 cm). Cathodic chamber contained 6.371 g K_3_Fe(CN)_6_ (potassium ferricyanide; BAKER ANALYZED, A.C.S. Reagent) and 17.429 g K_2_HPO_4_ (dipotassium hydrogen phosphate; SHOWA Co. Ltd.) well dissolved in 200 mL deionized-distilled water [44].

### Microbial cultures

In this work, LB medium used in MFCs provided essential nutrient sources for bacterial growth and metabolic functions (e.g., electron-transport capability). The electroactive bacteria *Shewanella haliotis* WLP72 and *Aeromonas hydrophilia* NIU01 (originally isolated from azo dye decolorization as the selection pressure) were used for bioelectricity generation in MFCs operated at ambient temperature (ca. 25 °C).

### Preparation of anthocyanin-related plant extracts

To obtain plant extract samples, *Lycium ruthenicum* Murr. (*L. ruthenicm* Murr., commonly known as “black wolfberry”), *Clitoria ternatea* Linn. (*C. ternatea* Linn., commonly known as “butterfly pea”) and *Vaccinium* Spp. (commonly known as “blueberry”) at 5 g were first freeze-dried and then ground into powders. The freeze-dried powders were extracted with 100 mL of methanol/water/acetic acid (85:15:0.5, v/v, MeOH/H_2_O/AcOH) as described elsewhere [45]. After refrigeration under the condition of − 80 °C overnight, frozen extract were placed into the freezer dryer for 48 h and then pulverized. The resulting powder was possibly light-sensitive and then placed in brown drying containers to avoid deliquesce and illumination.

### Cyclic voltammetric measurement

Cyclic voltammetry of extract samples was carried out via a work station for electric chemistry analysis (Jiehan 5600, Jiehan Technology Corporation, Taiwan). A glassy carbon electrode (0.07 cm^2^; CH Instruments Inc., SA) polished with 0.05 μm alumina polish was used as the working electrode. Quadrate platinum electrode (6.08 cm^2^) served as the counter electrode and was soaked in hydrogen peroxide (H_2_O_2_) prior to use. As the reference electrode, a Hg/Hg_2_Cl_2_ electrode was filled with saturated KCl_(aq)_ to keep the stability and reproducibility. Prior to analysis, the test solutions were inevitably purged with nitrogen for 15 min to removal residual oxygen. The symmetric scan range from − 1.5 to + 1.5 V were carried out with a scanning rate of 10 mV s^−1^. As the direct parameter to assess the redox capacity, closed curve area of redox potential $\left( {{\text{i}}.{\text{e}}., {\text{Area}} = \mathop \smallint \nolimits_{{V_{\text{L}} }}^{{V_{\text{H}} }} \left( {i_{\text{h}} - i_{\text{l}} } \right){\text{d}}V} \right)$ were calculated with Origin 8. In this way, *V*_H_, *V*_L_ represented the CV scanning voltages of + 1.5 V and − 1.5 V, respectively; *i*_h_, *i*_l_ presented the oxidation currents and the reduction currents at specific scan voltage, respectively. Moreover, 100 cycles of CV scan were conducted to verify the reversibility and stability of redox characteristics.

### Antioxidant activity analysis

Regarding antioxidant activity, the DPPH free-radical (C_18_H_12_N_6_O_5_; 2,2-diphenyl-2-picryl hydrazyl) scavenging activities were usually used as the standard assessment methods. Also, the DPPH free radical scavenging rate, as the key parameter to represent antioxidant activity, was determined via the formula:

$${\text{DPPH radical scavenging}} = \left( {1 - \frac{{{\text{Abs}}_{\text{sample}} - {\text{Abs}}_\text{{blank}} }}{{{\text{Abs}}_\text{{control}} }}} \right) \times 100 .$$

The chemical reaction between DPPH free radical and the test samples were kept at 30 min. During this process, Abs_sample_ (DPPH + sample), Abs_blank_ (ethanol solvent + sample; 2:1/v:v) and Abs_control_ (DPPH + ethanol solvent; 1:2/v:v) were determined via a spectrophotometric analyzer (GENESYS 10S UV–Vis) with a detecting wavelength of 515 nm [46].

### Determination of total polyphenolics content (TPC)

According to Naczk and Shahidi [47], TPC of anthocyanin-rich extract was quantitatively determined as follows: First, Folin-Ciocalteu reagent (1:1/v:v with deionized water), test samples (redissolution to 1000 mg L^−1^) and saturated sodium carbonate (Na_2_CO_3_) were prepared prior to use. Then, these three solutions were mixed in 4 mL of deionized water with the amount of 0.25 mL, 0.25 mL and 0.5 mL. After chemical reaction for 25 min at room temperature, the mixture was centrifuged to remove particulates and obtain the supernatant. The absorbance of mixture supernatant was determined via a spectrophotometric analyzer (GENESYS 10S UV–Vis) at maximal absorption wavelength of 725 nm. TPC content was determined by gallic acid as the standard and thus expressed in terms of GA equivalents (GAE) in extract weight (EW).

### Electrochemical measurements

Transient voltage was automatically collected with a data acquisition system (DAS 5020; Jiehan Technology Corporation, Taiwan). For comparison with prior results, the external resistance of microbial fuel cells was set to 1 KΩ. Power density and current density of MFCs were calculated with the formulae as shown below:

$$P_{\text{density}} = \frac{{V_{\text{MFC}} \times I_{\text{MFC}} }}{{A_{\text{anode}} }},$$

$$I_{\text{density}} = \frac{{I_{\text{MFC}} }}{{A_{\text{anode}} }},$$

where *V*_MFC_ and *I*_MFC_ could be directly measured with linear sweep voltammetry supported by a work station for electric chemistry analysis (Jiehan 5600, Jiehan Technology Corporation, Taiwan). *A*_anode_ represented for the actual working area of the anode.

### Adenosine triphosphate detection

The bioenergy content of bacteria during bioelectricity generation was also detected with an ATP-bioluminescence detector (LBY-420, lvbang-tech) based on luciferin/luciferase reaction. The bioluminescence technique was based on the light-producing organism-firefly luminescence principle, which rapidly abd effectively detected ATP with luciferase-fluorescein system. Here, ATP was a limiting factor of the bioluminescent reaction. Luciferin substrate, as the light-carrier substance or the energy-transfer substance, played an indispensability role on the luminescent system of luciferase in the bioluminescence. In addition, the integrated light intensity was directly proportional to ATP contents for ATP-bioluminescence using luciferin/luciferase. Before and after the supplementation of anthocyanin-rich extract into MFCs, the detection bar with fluorescein was immersed and then fast inserted into the detector. The ATP content could be transformed into visible signal through fluorescence reaction within 20 s.

## Abbreviations

RMs: redox mediators
MFCs: microbial fuel cells
ATP: adenosine triphosphate
ET: electron transfer
GA: gallic acid
Dp: delphinidin
Pg: pelargonidin
Cy: cyanidin
Pn: peonidin
Pt: petunidin
Mv: malvidin
CV: cyclic voltammetric
TPC: total polyphenolics content
GAE: GA equivalents
EW: extract weight
ADP: adenosine diphosphate
AMP: adenosine monophosphate
